# Biocontrol potential of endophytic bacteria against *Fusarium* wilt pathogen (*Fusarium* *oxysporum* f. sp. *ciceris*) in chickpea

**DOI:** 10.3389/fmicb.2026.1787705

**Published:** 2026-05-28

**Authors:** Jonnada Likhita, Meenakshi Arya, Yashowardhan Singh, Maharishi Tomar, Prashant P. Jambhulkar, Anshuman Singh, Vister Joshi, Manoj Choudhary, Usha, Sushil K. Chaturvedi

**Affiliations:** 1 Department of Plant Pathology, Rani Lakshmi Bai Central Agricultural University, Jhansi, Uttar Pradesh, India; 2 ICAR-National Research Institute for Integrated Pest Management, New Delhi, India; 3 ICAR-Central Institute of Post Harvest Engineering and Technology, Ludhiana, Punjab, India; 4 ICAR-Indian Agricultural Statistics Research Institute, New Delhi, India; 5 Krishi Vigyan Kendra, Jalaun, India

**Keywords:** 16S rRNA sequencing, biochemical, biological control, chickpea, endophytic bacteria, *Fusarium oxysporum*

## Abstract

**Introduction:**

*Fusarium wilt* of chickpea, caused by *Fusarium oxysporum* f. sp. ciceris (FOC), is a major soil-borne disease that severely affects chickpea production worldwide. The present study aimed to isolate and characterize endophytic bacteria from different plant species and evaluate their antagonistic potential against FOC.

**Methods:**

Endophytic bacteria were isolated from leaves, stems, and roots of various plant species. A total of 73 bacterial isolates were obtained from 210 plant samples and screened for antagonistic activity against FOC under *in vitro* and *in vivo* conditions. Five promising isolates were selected for detailed studies, including biochemical characterization, stress tolerance assays (salinity, heat, and drought), and molecular identification through 16S rRNA gene sequencing.

**Results:**

The selected isolates were identified as *Ochrobactrum quorumnocens, Bacillus velezensis, Alcaligenes faecalis, Bacillus subtilis*, and *Acinetobacter schindleri*. These isolates exhibited significant inhibition of FOC on potato dextrose agar (PDA), with inhibition percentages of 36.29%, 48.48%, 45.89%, 57.37%, and 55.50%, respectively. Under field conditions, all five isolates significantly reduced disease incidence and enhanced chickpea growth compared to the untreated control.

**Discussion:**

The findings demonstrate that the selected endophytic bacterial isolates possess strong antagonistic activity against FOC and also exhibit plant growth-promoting traits. These isolates have considerable potential as biocontrol agents and PGPR for sustainable management of chickpea Fusarium wilt under both laboratory and field conditions.

## Graphical Abstract

**Figure:**
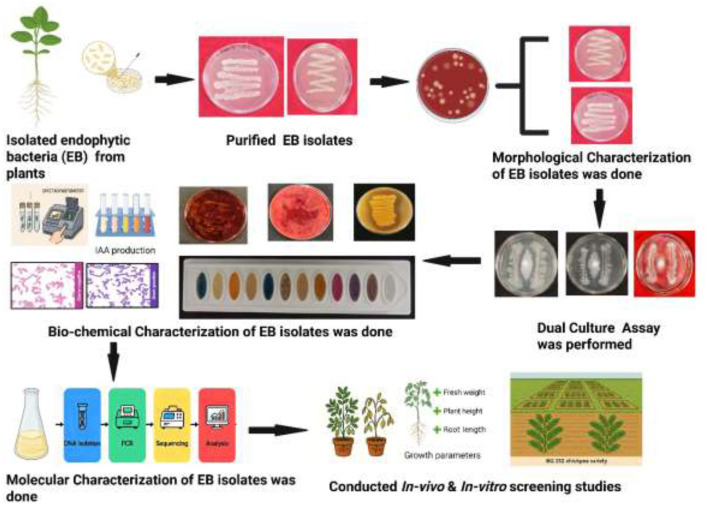

## Highlights

- 73 endophytic bacteria were isolated and screened against *Fusarium oxysporum* f. sp. *ciceris*.
- Five most potential isolates were identified by biochemical tests and 16S rRNA sequencing.
- The isolates showed significant *in-vitro* inhibition, with *Bacillus subtilis* being the most effective.
- Field evaluation confirmed disease reduction and growth promotion in chickpea.

## 1 Introduction

Chickpea (*Cicer arietinum* L.) stands in the third position in terms of the most important legume crops in the world, the first and second positions being occupied by common beans and field peas respectively. According to (Merga and Haji 2019), India dominates global chickpea production, contributing nearly two-thirds of the world's supply. Significant production also comes from Pakistan, Turkey, Iran, Myanmar, Australia, Ethiopia, Canada, Mexico, and Iraq. Unfortunately, chickpea production suffers from severe loss because of soil-borne fungal pathogens *Fusarium oxysporum* f. sp. *ciceris* (FOC), *Sclerotium rolfsii*, and *Rhizoctonia solani*, which incur tremendous economic losses each year (Mishra and Pandey, 2024). Generally, it is thought that *F. oxysporum* f. sp. *ciceris* is an obligatory facultative saprophyte that survives in soil and crop debris as chlamydospores for a very long time (up to 6 years) (Agrios, 2005) and is characterized by symptoms such as petiole drooping, wilting of the rachis, and discoloration in the xylem (Jendoubi et al., 2017). The degree of the disease and resulting crop yield loss (10–100 %) depends on aggressiveness, virulence of the fungal inoculum, and environmental conditions. In these days, conventional management strategies employ chemical fungicides as a great portion, but these products frequently harmfully affect non-target soil microbes or other dwelling organisms (Singh et al., 2016).

Endophytic bacteria (EB) are defined “as those that grow in tissues of plants without causing any ill health on their hosts”. The actual potential use of these live endophytes in plants was studied for the perusal of plant growth enhancement and biological disease control (Xia et al., 2022; El-Saadony et al., 2022) through better nutrient uptake, through phyto-hormones synthesis, degradation of contaminants, improved defences against plant pathogens (Bent and Chanway, 2002). Examples include *Bacillus, Pseudomonas, Enterobacter, Micrococcus, Acinetobacter, Azotobacter*, and *Azospirillum* that are known for their siderophore production, phosphate, potassium solubilizers, and antibiotic action, hydrogen cyanide, and immune-suppressant excretors that hinder phytopathogens (Timofeeva et al., 2023; Rochlani et al., 2022) They are characterized through molecular means such as 16S rDNA sequencing or detection of the nifH gene to post-identification of these bacteria from different crops *viz.*, sugarcane, rice, tomato, and medicinal plants (Debasis et al., 2019; Duhan et al., 2020; Malviya et al., 2022).

Strategies to control the wilt complex in chickpea include crop rotations, field hygiene, planting or genetically engineering resistant varieties, soil solarization with amendments, bio-fumigation, and systemic acquired resistance (SAR) inducers (Sharma et al., 2024; Atwa and El Blasy, 2025). Endotoxins act against the fungi *F. oxysporum, S. rolfsii*, and *R. solani* which help to reduce disease severity by activating the induced systemic resistance (ISR) (Shanmugam and Kanoujia, 2011). Defence reactions in plants mainly involves two processes *viz.*, ISR and SAR in which ISR means that enhanced plants have the capacity of an intrinsic defence to biotic threats, whereas SAR means that the plants are granted long-term immunity (Sahu et al., 2020; Singh et al., 2020; Rakhonde et al., 2024). According to many research evidences, bio inoculants, including microbial biofertilizers and biopesticides, are sustainable alternatives to chemical-fertilizers Bala (2022). Microbial fertilizer as an alternative to chemical fertilizer in modern agriculture. In *Beneficial microorganisms in agriculture* (pp. 111–130). Singapore: Springer Nature Singapore (Bala, 2022). In the area of agriculture, endophytes are finding new and novel applications in the field of drug discovery and environmental remediation besides creating a better environment under which improvement of production in secondary metabolites takes place and increases the tolerance to stress (Anand et al., 2023). Globally, these efforts in such microbes harness their untapped potential for sustainable agriculture keeping in view their underutilized sources in controlling chickpea wilt complex.

They have undergone many changes during the last few decades; endophytic microorganisms having gained great recognition in agriculture and medical fields as well as in environmental pollution management. Beneficial endophytes are used as biofertilizers, biocontrol agents, or stress modulators with regards to agriculture, although their further application is present in the biopharmaceutical field because of their potential to synthesize bioactive compounds or nanoparticles, thus making them scanty resources in the health context as well (Sessitsch et al., 2004). New technologies connected to devising methods of environmental management techniques involving use of endophytes for the mitigation of hazardous industrial wastes or pollutants from soil are being developed (Digra and Nonzom, 2024). Generally, it refers to the microorganism existing within tissues of a host without any clear signs of infection. Such isolation and characterization would be done by the equipment of combined techniques involving “morphological, biochemical and molecular characterization of bacterial endophytes” of different plant species. It is consequently exciting to consider potential uses for control of *Fusarium* wilt in chickpeas through investigation of the involvement of endophytes with antimicrobial activity and consideration of some of the more significant root diseases such as the wilt complex that chickpea experiences. Endophytes in plants gain local as well as systemic protection by soil-borne fungal pathogens. Keeping this in mind, the present investigation was directed towards screening of strong antagonizing bacterial endophytes against FOC with a view to biocontrol of wilt disease in chickpea.

## 2 Material and methods

### 2.1 Collection and isolation of endophytic bacteria

A total of 210 plant samples were collected from the agricultural farm of RLBCAU, Jhansi, taking 2–3 samples per plant. In aseptic polyethylene bags, the samples were brought to laboratory conditions under transport at refrigerated conditions. Leaf, stem, and root tissues were washed with running tap water, surface sterilized with sterile distilled water (SDW) (1 min) and 1% sodium hypochlorite (4 min), and washed 3–4 times with SDW. 1 gm sterilized tissue was macerated in 10 ml SDW, and leaving it undisturbed for 15–20 min. Supernatant was diluted up to five folds and from the final dilution, 10 μL suspension was streaked onto NA plates and incubated at 28 ± 2 °C for 48h to isolate bacterial endophytes (Shah et al., 2021). Subsequent streaking of single colonies on LB agar plates allowed for additional purification before being preserved on LB slants at 4 °C for further investigations.

### 2.2 Screening of endophytic bacteria for its antagonistic effect against FOC

The experimental design for testing antagonistic effect of isolated endophytic bacteria against FOC was done through a dual-culture assay as suggested by Singh et al. (2016) and Bhargavi et al. (2024). FOC was grown on PDA for 3 days at 28 ± 2 °C while bacterial isolates were cultured on NA for 24 h under similar conditions. A 5 mm FOC mycelial plug was placed at the centre of a PDA plate and each endophytic bacterial isolate was streaked onto it while FOC-only plates served as controls. Plates were incubated at 28 ± 2 °C till the controls showed full growth of fungi. All treatment and control incorporating three replications. Incubation was done at 25 °C and hyphal morphology at colony edges was observed using a light microscope. Inhibition zones were measured according to the method of Sahu et al. (2020).


$$\begin{array}{l} \text{IR}\left(\text{\%}\right)=\frac{Control\;\left(C\right)-Treatment\;\left(T\right)}{Control\;\left(C\right)}\times \;100 \end{array}$$

### 2.3 Morphological and biochemical characterization of bacterial endophytes

Preliminary identification of endophytic bacteria (EB) was carried out based on their morphological and biochemical characteristics. Microscopic features, Gram reaction, and KOH test were assessed along with key biochemical assays including amylase, catalase, oxidase, cellulase, protease activity, phosphorus solubilization, motility, and H_2_S production. For morphological characterization, a purified single colony of each isolate was streaked onto Luria–Bertani (LB) agar plates and incubated at 28 °C for 72 h. Colony characteristics such as shape, size, colour, margin, and overall appearance were recorded following standard procedures described by Mahlangu and Tai (2022).

#### 2.3.1 Biochemical Characterization of bacterial endophytes

Five endophytic bacterial isolates were selected and subjected to investigate their biochemical nature. The Gram staining of putative endophytic bacteria was executed as described in Zhang et al. (2022). Bacterial smears were made, heat-fixed, stained with crystal violet, treated with iodine, decolorized with 95% alcohol, and counter-stained with safranin. Observations were done in air-dried smears using a Cilika compound microscope under 40X magnification. The KOH test below was carried out according to Dinata et al. (2021), mixing 3% KOH with the loop full of culture, when a slimy thread formed, it was taken as Gram-negative and, if not, Gram-positive. The enzymatic and functional tests were carried out for amylase on starch agar (appearance of halo after iodine, Singh et al., 2022), While Yuan et al. (2012) studied catalase activity using 3% H_2_O_2_, oxidase was determined by using the oxidase disc method on KB medium. SIM agar was used for motility and H_2_S production tests, wherein the spread of growth from the stab line indicated motility, and blackening indicated H_2_S production. Flooding with iodine demonstrated cellulase activity on CMC agar, whereas protease activity was determined by the formation of a halo on skim milk agar Singh et al., (2022). For the measurement of phosphate solubilization, Pikovskaya's medium was used, marking the phenomenon as halo formation, while IAA production (Abdelwahed et al., 2023) was monitored by Salkowski's reagent at 535 nm. More insight into the nitrogen fixation (Li et al., 2016) comes from exercising in nitrogen-free Ashby broth as well as Jensen agar. The five isolates (RLB E-1, RLB E-25, RLB E-30, RLB E-33, and RLB E-64) were also characterized by using the Hi-Media KB013 Enterobacteriaceae identification kit and the KB009 Hi-Carbohydrate™ kit. The above tests are based on alterations in pH and substrate utilization of their corresponding indicators, indicating colour changes thus aiding in the interpretation of the biochemical profiles.

### 2.4 Thermostability

The temperature stability of antifungal metabolites was evaluated following the method described by Myo et al. (2019). Five potent endophytic bacterial isolates were cultured in nutrient broth (NB) under agitation at 130 rpm and 28 °C for 48 h. After incubation, the culture filtrates were collected and subjected to different temperature treatments: 40 °C, 50 °C, 60 °C, 70 °C, 80 °C, and 90 °C, each for 30 min. The treated filtrates were then assessed for their antagonistic activity against *Fusarium oxysporum* f. sp. *ciceris* (FOC) using the dual culture assay.

### 2.5 Evaluation of endophytic strain tolerance to drought and salinity stress

Salt tolerance of potential bacterial isolates was assessed by streaking them on LB agar amended with 1–9% (1%, 3%, 5%, 7%, 9%) NaCl and incubating at 28 °C for 1–2 days. In addition, triplicate spot inoculation on nutrient agar plates with varying amounts of NaCl (0–9%) was performed by using overnight cultures and incubated for 3 days at 30 °C for their growth behaviour observation (Shah et al., 2021). Drought tolerance has also been measured as reported by Pirhadi et al. (2016) on isolates growing in Tryptic Soy Broth supplemented to 0–9 % PEG (1, 3, 5, 7, and 9%). During these assays, cultures having OD600 of 0.1 were incubated at 28 °C for 24 h. Drought-stress tolerance was then measured by OD600 readings post-incubation (Sandhya et al., 2017).

### 2.6 Molecular Identification of the most potential bacterial isolates

DNA was extracted using a protocol based on the method of Mahuku (2004). Bacterial cultures were grown to an OD600 of 0.4–0.5 at 30 °C, harvested by centrifugation, and washed with TE buffer. The cells were lysed by lysozyme, RNAse, Sodium dodecyl sulphate, and proteinase K. The proteins were removed by PCI extraction, followed by precipitation of DNA with ethanol, and washing with 70% ethanol, air drying, and storing in TE buffer at 4 °C for short-term storage. The amplification of the 16S rRNA was performed at an annealing temperature of 52.5 °C with primers 27F and 1492R (Hussain et al., 2023) in 10 μL reactions containing template DNA, Hi-Media Ready PCR Master Mix, primers, and sterile water. PCR was performed under the following conditions: initial denaturation at 94 °C for 4 min, followed by 35 cycles of 94 °C for 1 min, 52.5 °C for 1 min, and 72 °C for 1 min, ending with final extension at 72 °C for 10 min. The amplicons (~1,500 bp) were checked in 1% agarose gel and Sanger sequenced (Anuvanshiki Pvt. Ltd.). Sequence similarity search was performed using BLASTn, and the sequences were deposited to GenBank for accession numbers. For phylogenetic purposes, neighbour-joining analysis was performed using MEGA v12.

### 2.7 PCR detection of chitinase and lipopeptide antibiotics genes

Polymerase Chain Reaction (PCR) will be performed for the confirmation of chitinase and lipopeptide antibiotic genes present in the isolates. Out of these five strains, each of which has shown antifungal activity, the investigation will be conducted for the screening of chitinase and lipopeptide biosynthetic genes *viz.*, surfactin, iturin-A, fengycin-D, and bacillomycin-D. The primers used in amplifying the target genes have been presented in Table 1. PCR conditions for ChiA were the same as those described by Ramaiah et al. (2000): initial denaturation at 94 °C, 4 min followed by 35 cycles at 92 °C, 58 °C, and 72 °C, each 1 min, with a final elongation at 72 °C, 7 min. According to El-Hamshary et al. (2008), general chitinase gene amplification involved the following procedure: at 94 °C for 3 min, 30 cycles of 1-min denaturation at 94 °C, 1-min annealing at 50 °C, 1.5-min extension at 72 °C, followed by 7-min finishing at 72 °C. For lipopeptide gene amplification, the same conditions were applied as those according to Gond et al. (2015): 95 °C for 5 min and then 30 cycles of 94 °C, 1 min, 55 °C, 1 min, 72 °C, 1 min, with final extension of 72 °C, 10 min. All the PCR reactions had been done in triplicate to ensure reproducibility of results.

**Table 1:** PCR detection of “chitinase and lipopeptide” antibiotics genes.

Gene	Primers	Sequences	Amplicon size	References
Chi-A	Qchi-F	5-GATATCGACTGGGAGTTCCC-30	225	Ramaiah et al., 2000
	Qchi-R	5-CATAGAAGTCGTAGGTCATC-3		
Chitinase	Bchi-F	5-TTCAYGTTCAACACTACAA-3	310	El-Hamshary et al., 2008
	Bchi-R	5-CATTAAGGCCGCGGARTG-3		
Sfp	Sfp-F	5-ATGAAGATTTACGGAATTTA-3	675	Gond et al., 2015
	Sfp-R	5-TTATAAAAGCTCTTCGTACG-3		
Itu-D	ItuD-F	5-GATGCGATCTCCTTGGATCGT-3	647	
	ItuD-R	5-ATCGTCATGTGCTGCTTGAG-3		
Fen-D	FenD-F	5-TTTGGCAGCAGGAGAAGTTT-3	964	
	FenD-R	5-GCTGTCCGTTCTGCTTTTTC-3		
Bam-C	BamC-F	5-GAAGGACACGGAGAGAGTC-3	875	
	BamC-R	5-CGTGATGACTGTTCATGCT-3		

### 2.8 Assessment of antagonistic bacterial efficacy against *Fusarium* wilt in chickpea under pot and field conditions

Pot culture and field records monitored five endophytic bacterial isolates against *Fusarium oxysporum* f. sp. *ciceris* in chickpea. Pot experiments (*Rabi* 2023–24 and 2024–25) were conducted using moderately resistant variety BG-212. The bacterial inoculum, maintained for 48 h in nutrient broth (28 ± 2 °C), was adjusted to 108 CFU/ml, and surface-sterilized seeds were soaked for 12 h in EB suspensions or in sterile water (control) (Akram et al., 2024). Fifteen seeds treated were then sown per pot with sterilized soil. At 10 days after germination, 100 ml of bacterial suspension was drenched, followed by pathogen inoculation after 15 days of sowing with *F. oxysporum* infested wheat grains. Twenty treatments were laid down in a CRD with three replications and parameters recorded were germination and wilt-related mortality (Senthilkumar et al., 2009). Simultaneously, field trial was conducted in the wilt-affected field plot with BG-212 (Block D22, RLBCAU, Jhansi) laid down in a randomized block design with 19 treatments replicated thrice. Germination percentage, disease incidence, and yield were recorded at 15, 30, and 45 DAS, which were calculated according to the protocols given by Samjik, 2024. Statistical analysis was performed in OPSTAT.

## 3 Results

### 3.1 Isolation of bacterial endophytes

**Figure 1:**
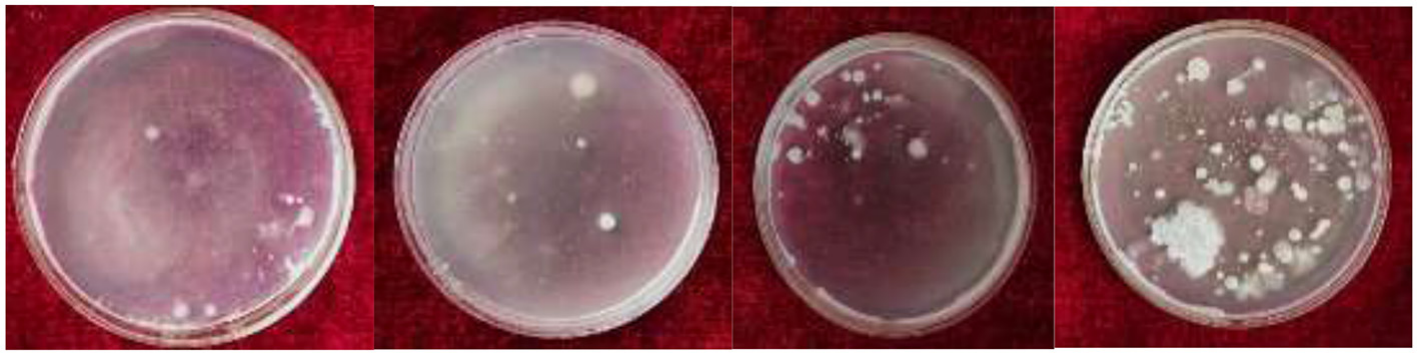
Isolation of endophytic bacteria by serial dilution method.

**Figure 2:**
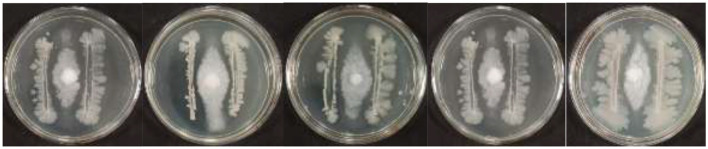
Purified endophytic bacterial isolates.

Samples from 13 different plants (*viz.*, rice, chickpea, taro, milkweed, lawn grass, moong bean, spinach, bean, mustard, okra, tomato, onion, and wheat) were brought to the lab and used for the isolation of endophytic bacteria. A total of 73 bacterial isolates were isolated from 210 different plant parts of these samples and were further sub-cultured on NA plates (Figures 1, 2). Later, the isolated endophytic bacteria (EB) were labelled as RLB E-1 to RLB E-73 (Supplementary Table 1). These endophytic bacterial isolates were then assessed for their antagonistic activity against FOC.

### 3.2 Antagonistic activity of effective endophytic bacteria against FOC

**Figure 3:**
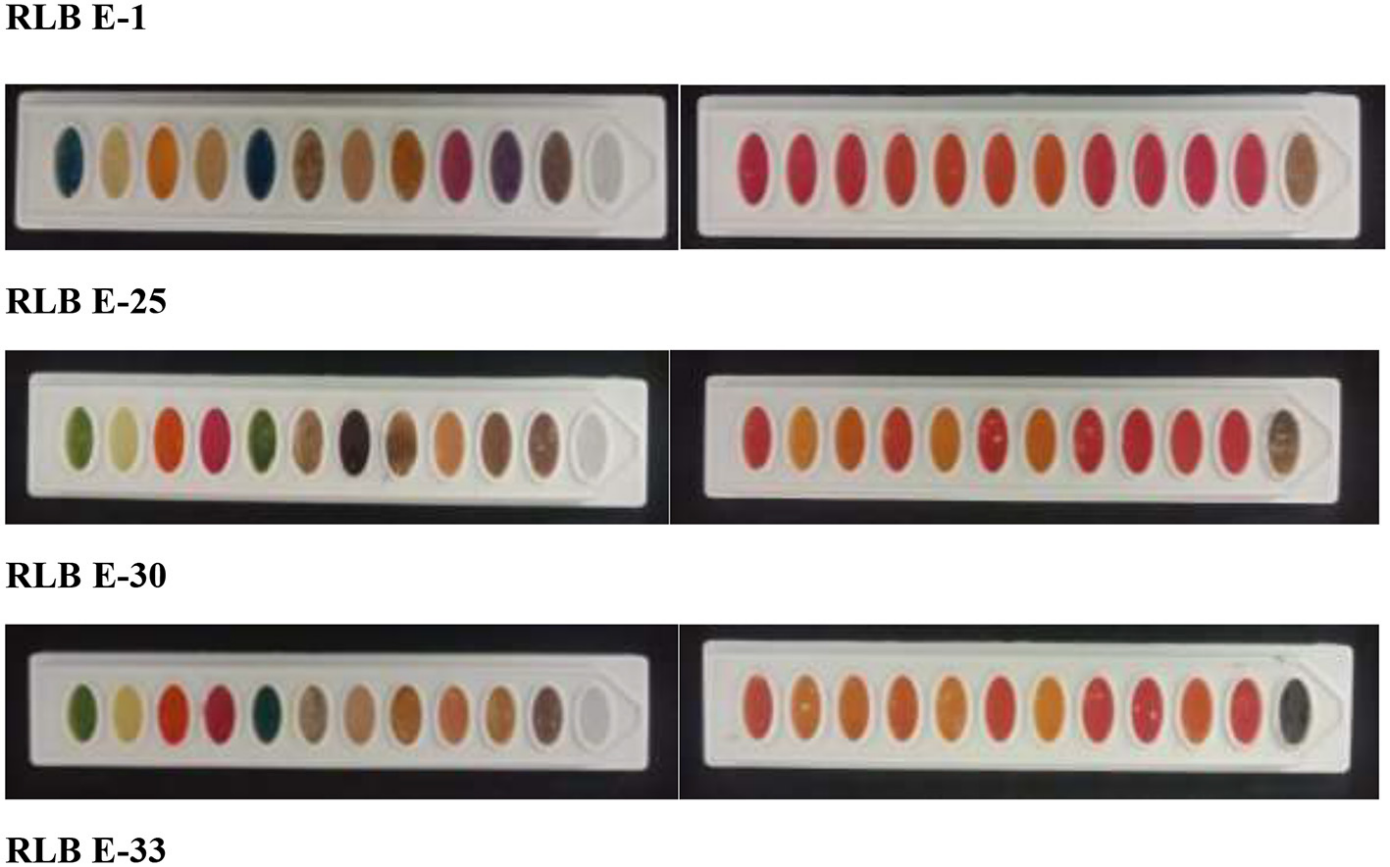
Screening of endophytic bacteria for its antagonistic effect against FOC.

Five endophytic bacteria were tested antagonistically against FOC via dual culture assay (Table 2). Control plates exhibited maximum radial growth of 71.16 mm, whereas the treatments showed restricted growth ranging from 30.33 to 45.33 mm. Among the isolates, RLB E-33 was the most effective in restricting radial growth to 30.33 mm, resulting in the highest inhibition of 57.37%, followed by RLB E-64 with 55.50% inhibition. Two isolates, *viz.*, RLB E-25 and RLB E-30, also demonstrated considerable antagonistic activity with inhibition percentages of 48.48% and 45.89%, respectively. In contrast, RLB E-1 exhibited the least inhibitory effect (36.29%) with a radial growth of 45.33 mm. The statistical analysis [CD at 5% = 3.89; SE(m) = 1.25] indicates significant differences among the isolates. These findings highlight RLB E-33 and RLB E-64 as the most promising antagonists for suppressing the growth of the test pathogen (Figure 3).

**Table 2:** Antagonistic activity of effective endophytic bacteria against FOC.

Isolate	Radial growth (mm)	% inhibition
RLB E-1	45.33	36.29
RLB E-25	36.66	48.48
RLBE-30	38.50	45.89
RLBE-33	30.33	57.37
RLBE-64	31.66	55.50
Control	71.16	
C.D. (5%)	3.89	
SE (m)	1.25	

### 3.3 Morphological characterization of effective endophytic bacterial isolates

Pure single isolated and purified bacteria colonies were streaked on King's B (KB) media and incubated after that at 28 °C for 72 h. The shape, size and colour, margin, and elevation of such colonies were studied (Supplementary Table 2). Based on those characteristics and combined observation with the result of Gram staining, five bacterial isolates-RLB E-1, RLB E-25, RLB E-30, RLB E-33, and RLB E-64 were selected for the next characterization following established procedures. Among them, 4 isolates-RLB E-1, RLB E-30, RLB E-33, and RLB E-64, formed round colonies with smooth margins. However, RLB E-25 formed irregular-shaped colonies with rough margins. The colony size of all isolates varied from small to large, being comparatively larger in the case of RLB E-25 with respect to others. All the isolates were creamish pigmented, a trait characteristic of rhizosphere inhabiting bacteria. Based on Gram staining, three isolates-RLB E-1, RLB E-30, and RLB E-64-were classified as Gram-negative while RLB E-25 and RLB E-33 were positive. Hence there could be a mixed population of Gram-positive and Gram-negative bacteria in the endophytic bacterial community.

### 3.4 Biochemical characterization of endophytic bacterial isolates

The five endophytic bacterial isolates, namely RLB E-1, RLB E-25, RLB E-30, RLB-E33, and RLB E-64, varied in their biochemical and functional tests (Supplementary Table 3 and Supplementary Figure 1). For example, RLB E-1 reacts as Gram-negative and KOH positive, showing positive results for catalase, oxidase, and motility, with hydrogen sulphide (H_2_S) production being its negative attribute. On the contrary, it is negative for amylase, protease, cellulase, chitinase, and phosphate solubilization. It is worth noting that it had strong indole-3-acetic acid (IAA) production (0.886), suggesting a possible role in promoting plant growth by indole-3-acetic acid production via auxin. RLB E-30 is another Gram-negative bacterium that tested positive for KOH, it also showed positive tests for amylase, protease, motility, and H_2_S production while being negative for catalase, oxidase, phosphate solubilization, cellulase, chitinase, and IAA production with a low IAA level of 0.018. RLB E-33 was Gram-positive and KOH-negative. It tested positive for amylase, catalase, oxidase, protease, phosphate solubilization, motility, and H_2_S production, but it was negative for IAA, cellulase, and chitinase activity and had low IAA production (0.042). RLB E-64 was Gram-negative, KOH positive, and tested positive for amylase, catalase, oxidase, protease, phosphate solubilization, motility, and H_2_S production but negative for cellulase, chitinase, and very low IAA production (0.009). Thus, all isolates were motile and positive for H_2_S production but negative for cellulase and chitinase. RLB E-1, however, was rated the best possible isolate with IAA production, while RLB E-25, RLB E-33, and RLB E-64 were very good in different enzymatic and plant growth-promoting traits such as amylase, catalase, oxidase, and phosphate solubilization.

### 3.5 Biochemical characterization by Hi-Media IMVIC tests

**Figure 4:**
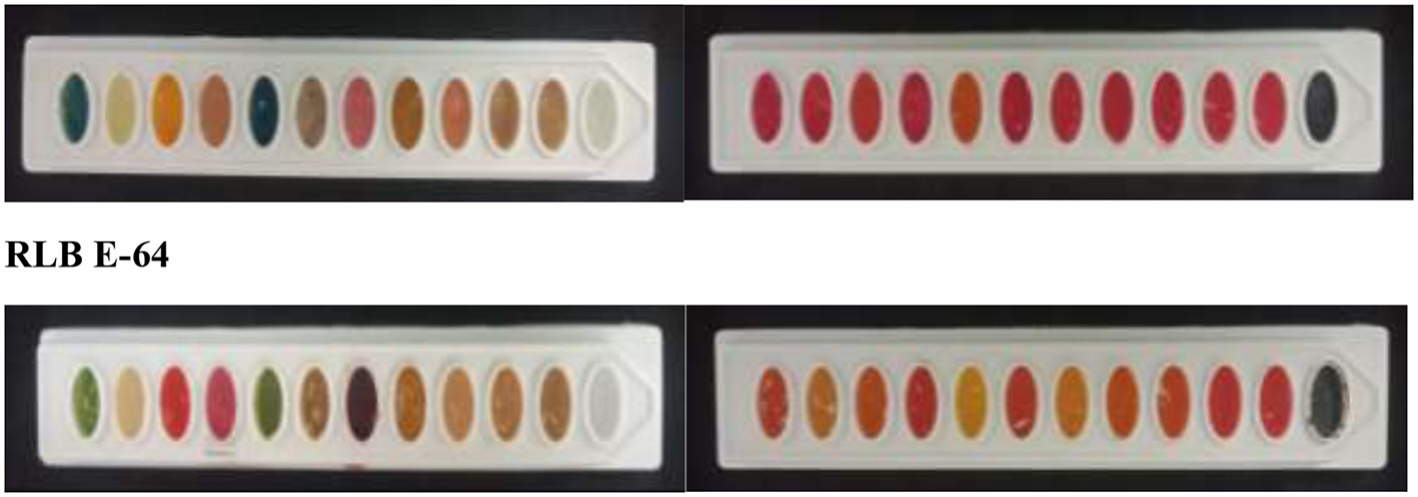
Biochemical tests by HiMedia test.

Biochemical profiling of the endophytic bacterial isolates showed completely different metabolic abilities with respect to the tested parameters (Supplementary Table 4 and Figure 4). All endophytic bacterial tested for ONPG, phenylalanine deamination, indole production, and lactose fermentation were negative which means absence of β-galactosidase activity, deaminase activity, tryptophanase, and lactose-utilizing pathways. The metabolizing for amino acid showed that RLB E-1, RLB E-25, RLB E-30 were lysine decarboxylating, only RLB E-1 and RLB E-25 were positive for ornithine utilization. The production of urease was revealed by the strong reaction of RLB E-1, with RLB E-33 also being positive, while all other isolates failed to show urease production. Nitrate is reduced by RLB E-25, RLB E-33, and RLB E-64 isolates. Only RLB E-33 produced the H_2_S, and citrate utilization having been recorded in RLB E-1, RLB E-30, and RLB E-33. Strong positives for VP were in RLB E-25, RLB E-30, and RLB E-64 as well as moderate in RLB E-33. Mixed-acid fermentation was indicated by Methyl Red-positive RLB E-25, RLB E-30, and RLB E-64. In the use of malonate, RLB E-1, RLB E-33 were positive. Hydrolysis of esculin was positive for all isolates, greater reactions in RLB E-30, RLB E-33, and RLB E-64. The patterns of carbohydrate fermentation were different for all isolates. RLB E-1 fermenting cellobiose and sucrose, RLB E-25 fermenting trehalose, RLB E-30 fermenting esculin, sucrose, and trehalose while RLB E-33 fermenting esculin and trehalose and RLB E-64 showing broad activity with adonitol, cellobiose, sucrose, and trehalose. Trehalose fermentation was positive for 4 isolates except RLB E-1. Glucose fermentation occurred only in RLB E-25, RLB E-30, and RLB E-64. Finally, all the isolates except RLB E-30 proved positive for oxidase activity, thus indicating aerobic respiratory capability in most of them.

### 3.6 Thermostability

Thermotolerance assessment of the five endophytic bacterial isolates 40–90 °C indicated that they differed greatly in terms of with stand to the upper limit of temperature (Supplementary Table 5). All the isolates showed moderately good growth at 40 °C, with RLB E-30 recording the highest. Growth increased at 50 °C for most isolates with RLB E-30, RLB E-1 and RLB E-64 thrived well. All isolates were capable of exhibiting considerable growth at 60 °C, with RLB E-30 recording again the highest level of tolerance. Growth was reduced at 70 °C except for RLB E-30, which exhibited greater inhibition even at these high temperatures. At 80 °C, the isolates showed varied responses: RLB E-25 and RLB E-33 has shown good inhibition. Surprisingly, all isolates endured even 90 °C, and RLB E-1 and RLB E-33 had the most growth, indicating special endurance capacity to high temperature. The fact that SE(m) values were quite low (0.41–0.49) aligned with results. In summary, RLB E-30, RLB E-1, and RLB E-33 were the most heat-tolerant among the above-mentioned isolates, rendering them ideal candidates for use under stress conditions from high-temperature environments.

### 3.7 Evaluation of endophytic strain tolerance to drought and salinity stress

The salt-tolerance assays have established that there are separate endophytic bacterial isolates that respond quite differently to salinity and drought (Supplementary Figure 2). In salt stress, RLB E-33 exhibited the best tolerance, as there was growth at levels of 7–9% NaCl; thus, it's adapted to salinity. RLB E-25 and RLB E-1 possessed lower tolerance levels but yet maintained growth at higher salt levels. On the contrary, the majority effects of salt increased for RLB E-30 and RLB E-64 with no or negative growth under salt conditions. Under drought (PEG treatments), the endophyte that possessed the highest level of tolerance was that of RLB E-30, maintaining OD600 comparable to or above that of control at 1–5%, and with reasonable growth rate even at 9%, showing a high adaptation to osmotic stress. RLB E-33 and RLB E-25 displayed fair to moderate resistance to drought, with RLB E-33 being the best performer at 3% PEG, while a prominent increase in growth was shown by RLB E-25 at 9% PEG. Across all PEG concentrations, RLB E-64 recorded generally low growths, indicating drought intolerances. RLB E-1 showed irregular and generally fluctuating growth under PEG-induced stress. Overall results indicate that RLB E-33 is the most salt-tolerant, while RLB E-30 was drought tolerant among the isolates, whereas RLB E-64 and RLB E-1 were the most stress-sensitive ones.

### 3.8 Molecular identification of the most potential endophytic bacterial isolates

The analysis of sequences of isolates displayed high homology with closely related bacterial species (Table 3). Identification of the bacterial isolates was carried out by means of 16S rRNA gene sequence analysis, and the sequences obtained were compared with reference sequences available from the NCBI GenBank database for an accurate taxonomic identification. The isolate RLB E-1 has been identified as *Ochrobactrum quorumnocens*, with the NCBI accession number PX710924.1 meanwhile, RLB E-25 was found to be completely similar to *Bacillus velezensis* (PX711159.1). By putting it down similarly, RLB E-30 was identified as *Alcaligenes faecalis* (PX711247.1), RLB E-33 as *Bacillus subtilis* (PX710790.1), and RLB E-64 as *Acinetobacter schindleri* (PX711283.1). All the isolates showed 100 per cent query coverage and 100 per cent sequence identity, stating that the entire sequenced region aligned perfectly to the respective reference sequences confirming precise species-level identification. The successful deposition of these sequences in the GenBank database substantiated with their assigned accession numbers lends additional credence to the authenticity and reliability of molecular identification. These bacteria isolates, which have been accurately identified, thus represent a solid starting point for their further study in plant growth promotion and disease management.

**Table 3:** Molecular identification of the most potential endophytic bacterial isolates.

Isolate code	Identified organism	Query cover	Percent identity	NCBI accession no.
RLB E-1	*Ochrobactrum quorumnocens*	100	100	PX710924.1
RLB E-25	*Bacillus velenzensis*	100	100	PX711159.1
RLB E-30	*Alcaligens faecalis*	100	100	PX711247.1
RLB E-33	*Bacillus subtilis*	100	100	PX710790.1
RLB E-64	*Acinetobacter schindleri*	100	100	PX711283.1

### 3.9 PCR detection of chitinase and lipopeptide antibiotics genes

Molecular screening of the endobacterial isolates revealed variability in the presence of antifungal and plant-beneficial genes. The Supplementary Table 6 illustrates the presence or absence of specific antibiotic and lipopeptide biosynthetic genes in different endophytic bacterial isolates (RLB E-1, RLB E-25, RLB E-30, RLB E-33, and RLB E-64). The Chit-A gene, involved in chitin degradation and important for antifungal activity, was detected in isolates RLB E-25, RLB E-30, RLB E-33, and RLB E-64, indicating their potential for breaking down fungal cell walls. However, none of the isolates possessed the Chitinase gene, suggesting that direct chitinase enzyme production might be absent or limited in these strains. Among the lipopeptide genes, Surfactin, known for its antimicrobial and biosurfactant properties, was present only in RLB E-33. The IturinD gene, which produces lipopeptides with strong antifungal effects by damaging fungal membranes, was detected only in RLB E-25. Interestingly, Fengycin D, typically effective against filamentous fungi, was not present in any of the isolates. The BamC gene, associated with outer membrane protein assembly and possibly contributing to stress resistance or antimicrobial properties, was observed in RLB E-1, RLB E-25, RLB E-30, and RLB E-64. Overall, isolate RLB E-25 exhibited the highest diversity of biocontrol-related genes, carrying ChitA, IturinD, and BamC, making it a strong candidate for biological disease management. RLB E-33 also showed promotable performance due to the presence of Surfactin and ChitA, while RLB E-1 had limited biosynthetic potential, with only BamC detected.

### 3.10 Assessment of antagonistic bacterial efficacy against *Fusarium* wilt in chickpea under pot and field conditions

In both pot and field experiments conducted over two seasons, the treatments displayed distinct differences with respect to seed germination, disease incidence, seedling mortality, and crop performance against *Fusarium oxysporum* f. sp. *ciceris* (Supplementary Figure 3). Under pot conditions (Supplementary Tables 7, 8), germination was quite variable, ranging from practically none for the single priming treatments (22.22% in T7 during Season-1 and 49.89% in T9 during Season-2) to consistently high for the combined seed priming and drenching treatments, especially T16 (RLB E-33), T18, and T20, which had 90% and more germination in both seasons. Less disease incidence (0.00–6.67% in T16; 8.89–15.55% in T18; 4.45–8.89% in T20) and minimum seedling mortality (13.48–16.46%), obtained by combined treatments, proved distinctly superior against their respective single priming or single drenching treatments, which assumed much higher values for disease intensity and mortality. Overall, T16 and the chemical control T20 gave excellent results in pot trials by effectively suppressing wilt and enhancing seedling survival. Similarly, field evaluation data demonstrated significant variations among treatments (Supplementary Table 9). Disease intensity recorded at 15, 30, and 45 DAS showed that treatment groups T6, T15, T13, and T4 had been consistently good in keeping infection levels low, while treatments T17, T18, and T19 performed poorly, yielding similarly high disease pressure to that of untreated control. Germination in the field ranged from 51.53% in the control to 94.76% in T15, where T15, T13, T14, and T6 came out on top by virtue of improved seed vigour and lesser pathogen interference. Yield data equally reflected this trend, with T15 coming out with the highest productivity (3.22 t ha^−1^) and control with the lowest yield (1.47 t ha^−1^). Results of both the pot and field studies, in general, corroborate that integrated application of seed priming plus drenching has significantly far better implications than either treatment on its own. Treatments such as T16 (E-33 RLB), T18, T20 in pot conditions and T15, T13, T14, T6 in field conditions have good disease suppression with enhanced germination, better seedling establishment, and high yields, thus showing promise as sustainable and efficient management options for chickpea wilt.

## 4 Discussion

Among the natural factors of production, plant diseases are major limitations to chickpea production on a global front. Chemical pesticides were the major control strategy used since time immemorial, but their continuous and indiscriminate use led to environmental pollution, resistance among pathogens, and harmful residues in food (Harish et al., 2008; De Curtis et al., 2010; Ma et al., 2015; Yang et al., 2015). Growing concern over food safety and sustainable agriculture has thus led to a search for less toxic alternatives (Souto et al., 2021: Leskovac and Petrović, 2023). Biological control using beneficial microorganisms has evolved as one of the best alternatives environmentally safe and sustainable approaches for the management of soil-borne diseases (Alqahtani, 2025). In particular, endophytic bacteria are receiving growing interest because these organisms colonize internal plant tissues harmlessly while directly or indirectly providing protection to plants from pathogens.

The present investigation has isolated seventy-three endophytic bacterial isolates from healthy plant tissues collected from different hosts. In the first screening step, by the dual-culture assay against FOC, five isolates (RLB E-1, RLB E-25, RLB E-30, RLB E-33, and RLB E-64) with potent antagonistic activity were identified, from which RLB E-25 and RLB E-33 were proved to have superior inhibition of the mycelial growth of FOC. The results have numerously correlated with previous findings from other crops (Das et al., 2023) and show that healthy plants harbour endophytic bacteria exhibiting antagonistic abilities against fungal pathogens (Bafana et al., 2008). The antagonistic isolates were characterized morphologically, biochemically, and molecularly. The isolates were identified based on 16S rRNA sequencing as *Ochrobactrum quorumnocens, Bacillus velezensis, Alcaligens faecalis, Bacillus subtilis, and Acinetobacter schindleri* while *Bacillus velezensis* (RLB E-25) and *Bacillus subtilis* (RLB E-33) displayed the strongest antagonism.

*Bacillus velezensis* and *Bacillus subtilis* have, thus far, been known for the promotion of plant growth (Jang et al., 2023), control of plant diseases (Wang et al., 2023) and the production of bioactive compounds like lipopeptides and enzymes (Ali et al., 2022). The study confirmed the presence of various biocontrol-related genes in these isolates, most notably ChitA, IturinD, Surfactin, and BamC involved with the synthesis of antifungal products and enzymes. Such genetic potential tends to account for the strong antagonistic performance for these isolates under *in vitro* conditions, as stated previously. The two isolates proved to possess PGPR traits, including IAA production, solubilization of phosphate, and protease and catalase activity, which indicated their multifunctional role in plant health improvement.

Further, pot culture and field validation for two consecutive seasons established that seed priming and soil drenching with these endophytic bacteria significantly improved seed germination, lessened disease intensity, and reduced pre- and post-emergence mortality compared to the untreated pathogen-inoculated controls. In particular, *B. velezensis* (RLB E-25) and *B. subtilis* (RLB E-33), which attained over 90 percent germination and the lowest disease incidence, were almost as effective as chemical fungicide treatment. In corroboration, there are similar reports stating that *Bacillus*-based biocontrol agents effectively suppress Fusarium wilt and enhance plant vigour (Nayyar, 2024). However, different control efficiencies between laboratory and field conditions are a common affair owing to environmental factors, soil conditions, inoculation methods, and means of colonization (Azarbad and Junker, 2024). On the other hand, the consistency in performance over the seasons may be indicative of their adaptability with great potential towards field application.

In conclusion, endophytic *Bacillus velezensis* (RLB E-25) and *Bacillus subtilis* (RLB E-33) have demonstrated antagonistic competence against FOC in this study as well as chickpea growth promotion under controlled and field conditions. Antifungal genes and stress tolerance traits detected in them further substantiate their biocontrol potential. Findings herein suggest that the endophytic isolates can emerge as green, environmentally friendly alternatives to chemical fungicides for managing *Fusarium* wilt in chickpea. Further study regarding metabolite profiling, colonization behavior, and formulation development would support the large-scale exploitation of these promising bacterial strains for sustainable agriculture.

## 5 Conclusion

Plant disease management should be sustainable and less unsafe. This means, where bacterial endophytes from natural sources are used, they serve as biocontrol agents instead of chemical pesticides. These naturally occurring helpful microbes can colonize the internal plant tissues without damaging them to form a mutually beneficial partnership with their hosts. In this regard, these microorganisms are biocontrol agents because they suppress plant pathogens through various mechanisms like production of antimicrobial metabolites, competition for nutrients and ecological niches, systemic resistance induction, cell-wall degrading enzyme secretion, which inhibit pathogen growth. Endophytic bacteria also help significantly augment plant growth besides pathogen suppression. They solubilize phosphates while fixing atmospheric nitrogen and enhance iron availability through the production of siderophores for nutrient uptake improvement. Numerous endophytes produce phytohormones such as IAA, gibberellins, and cytokinin's that stimulate root growth and generally pave way for plant vigour. Such a dual role of biocontrol and growth-promoting factors makes them invaluable for sustainable agriculture.

Increased restrictions on chemical pesticides, increased production costs, and rising consumer demand for safer and pesticide-free food have led pressure on the agriculture sector to move toward biological solutions to provide production alongside environmental safety. Bacterial endophytes would promise the best solution because they are compatible with plants, have low ecological risk, and could establish long-term beneficial interrelations within plant tissues. As research advances, development of endophyte-based bioinoculants, integrated into modern crop management systems, is predicted to become a possible element in the provision of sustainable-cum-climate-resilient agriculture with greater crop health and yield with the least negative impacts on the environment.

## Data Availability

The original contributions presented in the study are included in the article/Supplementary material, further inquiries can be directed to the corresponding author.

## References

[B1] Abdelwahed S. Cherif H. Bejaoui B. Saadouli I. Hajji T. Ben Halim N. . (2023). Microassay validation for bacterial IAA estimation as a new fine-tuned PGPR screening assay. Main Group Chem. 22, 143–154. doi: 10.3233/MGC-210124

[B2] Agrios G. N. (2005). Plant Pathology. Burlington, MA: Elsevier Academic Press.

[B3] Akram W. Waqar S. Hanif S. Anjum T. Aftab Z. E. H. Li G. . (2024). Comparative effect of seed coating and biopriming of Bacillus aryabhattai Z-48 on seedling growth, growth promotion, and suppression of Fusarium wilt disease of tomato plants. Microorganisms 12:792. doi: 10.3390/microorganisms12040792 38674736 PMC11052163

[B4] Ali N. Pang Z. Wang F. Xu B. El-Seedi H. R. (2022). Lipopeptide biosurfactants from *Bacillus* spp.: types, production, biological activities, and applications in food. J. Food Qual. 2022:3930112. doi: 10.1155/2022/3930112

[B5] Alqahtani F. S. (2025). The utilization of microorganisms for biological control of soil-borne plant pathogens: a sustainable strategy for managing plant diseases-a comprehensive review. J. Plant Pathol. 107, 1–25. doi: 10.1007/s42161-025-01984-1

[B6] Anand U. Pal T. Yadav N. Singh V. K. Tripathi V. Choudhary K. K. . (2023). Current scenario and future prospects of endophytic microbes: promising candidates for abiotic and biotic stress management for agricultural and environmental sustainability. Microb. Ecol. 86, 1455–1486. doi: 10.1007/s00248-023-02190-1 36917283 PMC10497456

[B7] Atwa M. A. El Blasy S. A. (2025). Induction of systemic acquired resistance against damping-off and stem rot diseases of chickpea caused by *Sclerotinia sclerotiorum*. EC Microbiol. 21, 1–15. doi: 10.1186/s41938-024-00837-w

[B8] Azarbad H. Junker R. R. (2024). Biological and experimental factors that define the effectiveness of microbial inoculation on plant traits: a meta-analysis. ISME Commun. 4:ycae122. doi: 10.1093/ismeco/ycae122 39507396 PMC11538580

[B9] Bafana A. Chakrabarti T. Devi S. S. (2008). Azoreductase and dye detoxification activities of *Bacillus velezensis* strain AB. Appl. Microbiol. Biotechnol. 77, 1139–1144. doi: 10.1007/s00253-007-1212-5 18034237

[B10] Bala K. (2022). “Microbial fertilizer as an alternative to chemical fertilizer in modern agriculture,” in Beneficial Microorganisms in Agriculture, eds. R. Prasad, and S.-H. Zhang (Singapore: SpringerNature Singapore), 111–130. doi: 10.1007/978-981-19-0733-3_4

[B11] Bent E. Chanway C. P. (2002). Potential for misidentification of a spore-forming *Paenibacillus polymyxa* isolate as an endophyte by using culture-based methods. Appl. Environ. Microbiol. 68, 4650–4652. doi: 10.1128/AEM.68.9.4650-4652.2002 12200326 PMC124109

[B12] Bhargavi G. Arya M. Jambhulkar P. P. Singh A. Rout A. K. Behera B. K. . (2024). Evaluation of biocontrol efficacy of rhizosphere dwelling bacteria for management of *Fusarium* wilt and *Botrytis* gray mold of chickpea. BMC Genom. Data 25:7. doi: 10.1186/s12863-023-01178-7 38225553 PMC10790480

[B13] Das S. Rabha J. Narzary D. (2023). Assessment of soil yeasts *Papiliotrema laurentii* S-08 and *Saitozyma podzolica* S-77 for plant growth promotion and biocontrol of *Fusarium* wilt of brinjal. J. Appl. Microbiol. 134:lxad252. doi: 10.1093/jambio/lxad252 37930719

[B14] De Curtis F. Lima G. Vitullo D. De Cicco V. (2010). Biocontrol of *Rhizoctonia solani* and *Sclerotium rolfsii* on tomato by delivering antagonistic bacteria through a drip irrigation system. Crop Prot. 29, 663–670. doi: 10.1016/j.cropro.2010.01.012

[B15] Debasis M. Snezana A. Panneerselvam P. Manisha C. Ansuman S. Vasić T. (2019). Plant growth promoting microorganisms (PGPMs) helping in sustainable agriculture: current perspective. Int. J. Agric. Vet. Sci. 7, 50–74. doi: 10.1016/b978-0-12-817004-5.00001-4

[B16] Digra S. Nonzom S. (2024). Bioremediation potential of endophytes: a promising tool. Appl. Biochem. Microbiol. 60, 694–714. doi: 10.1134/S0003683823602676

[B17] Dinata G. F. Aini L. Q. Kusuma R. R. (2021). Identification and characterization of antagonistic bacteria from coffee plant litter. Agrotechnol. Res. J. 5, 32–37. doi: 10.20961/agrotechresj.v5i1.49716

[B18] Duhan P. Bansal P. Rani S. (2020). Isolation, identification and characterization of endophytic bacteria from medicinal plant *Tinospora cordifolia*. S. Afr. J. Bot. 134, 43–49. doi: 10.1016/j.sajb.2020.01.047

[B19] El-Hamshary O. I. M. Salem H. H. Soliman N. A. (2008). Molecular screening of chitinase gene in *Bacillus* spp. J. Appl. Sci. Res. 4, 1118–1123. doi: 10.20546/ijcmas.2016.505.063

[B20] El-Saadony M. T. Saad A. M. Soliman S. M. Salem H. M. Ahmed A. I. Mahmood M. . (2022). Plant growth-promoting microorganisms as biocontrol agents of plant diseases: mechanisms, challenges and future perspectives. Front. Plant Sci. 13:923880. doi: 10.3389/fpls.2022.923880 36275556 PMC9583655

[B21] Gond S. K. Bergen M. S. Torres M. S. White Jr J. F. (2015). Endophytic *Bacillus* spp. produces antifungal lipopeptides and induce host defence gene expression in maize. Microbiol. Res. 172, 79–87. doi: 10.1016/j.micres.2014.11.004 25497916

[B22] Harish S. Kavino M. Kumar N. Saravanakumar D. Soorianathasundaram K. Samiyappan R. (2008). Bio-hardening with plant growth promoting rhizosphere and endophytic bacteria induces systemic resistance against Banana bunchy top virus. Appl. Soil Ecol. 39, 187–200. doi: 10.1016/j.apsoil.2007.12.006

[B23] Hussain A. Hasan A. Sherzada S. Noor T. Ahmad S. Kaoma M. . (2023). Bio-sorptive removal of selected metal ions from simulated wastewater using highly metal-resistant bacteria. Water Reuse 13, 448–458. doi: 10.2166/wrd.2023.059

[B24] Jang S. Choi S. K. Zhang H. Zhang S. Ryu C. M. Kloepper J. W. (2023). History of a model plant growth-promoting rhizobacterium, *Bacillus velezensis* GB03: from isolation to commercialization. Front. Plant Sci. 14:1279896. doi: 10.3389/fpls.2023.1279896 37885658 PMC10598611

[B25] Jendoubi W. Bouhadida M. Boukteb A. Béji M. Kharrat M. (2017). *Fusarium* wilt affecting chickpea crop. Agriculture 7:23. doi: 10.3390/agriculture7030023

[B26] Leskovac A. Petrović S. (2023). Pesticide use and degradation strategies: food safety, challenges and perspectives. Foods 12:2709. doi: 10.3390/foods12142709 37509801 PMC10379487

[B27] Li X. Geng X. Xie R. Fu L. Jiang J. Gao L. . (2016). The endophytic bacteria isolated from elephant grass (*Pennisetum purpureum* Schumach) promote plant growth and enhance salt tolerance of Hybrid Pennisetum. Biotechnol. Biofuels 9:190. doi: 10.1186/s13068-016-0592-0 27594917 PMC5010695

[B28] Ma X. Wang X. Cheng J. Nie X. Yu X. Zhao Y. . (2015). Microencapsulation of *Bacillus subtilis* B99-2 and its biocontrol efficiency against *Rhizoctonia solani* in tomato. Biol. Control 90, 34–41. doi: 10.1016/j.biocontrol.2015.05.013

[B29] Mahlangu S. G. Tai S. L. (2022). Morphological and molecular characterization of bacterial endophytes from *Centella asiatica* leaves. J. Genet. Eng. Biotechnol. 20:171. doi: 10.1186/s43141-022-00456-8 36576696 PMC9797633

[B30] Mahuku G. S. (2004). A simple extraction method suitable for PCR-based analysis of plant, fungal, and bacterial DNA. Plant Mol. Biol. Report. 22, 71–81. doi: 10.1007/bf02773351

[B31] Malviya M. K. Li C. N. Lakshmanan P. Solanki M. K. Wang Z. Solanki A. C. . (2022). High-throughput sequencing-based analysis of rhizosphere and diazotrophic bacterial diversity among wild progenitor and closely related species of sugarcane (*Saccharum* spp. inter-specific hybrids). *Front. Plant Sci*. 13:829337. doi: 10.3389/fpls.2022.829337 PMC8908384 35283913

[B32] Merga B. Haji J. (2019). Economic importance of chickpea: production, value, and world trade. Cogent Food Agric. 5:1615718. doi: 10.1080/23311932.2019.1615718

[B33] Mishra R. K. Pandey S. (2024). “Major fungal diseases of pulse crops and their management to mitigate its losses,” in Diseases of Field Crops: Diagnostics and Management, (Singapore: Springer Nature Singapore), 279–294.

[B34] Myo E. M. Liu B. Ma J. Shi L. Jiang M. Zhang K. . (2019). Evaluation of *Bacillus velezensis* NKG-2 for bio-control activities against fungal diseases and potential plant growth promotion. Biol. Control 134, 23–31. doi: 10.1016/j.biocontrol.2019.03.017

[B35] Nayyar N. A. (2024). Investigating the efficacy of biocontrol agents against root rot diseases in tomato crops. Indus J. Anim. Plant Sci. 2, 43–56. doi: 10.20546/ijcmas.2019.811.082

[B36] Pirhadi M. Enayatizamir N. Motamedi H. Sorkheh K. (2016). Screening of salt tolerant sugarcane endophytic bacteria with potassium and zinc for their solubilizing and antifungal activity. Biosci. Biotechnol. Res. Commun. 9, 530–538. doi: 10.21786/bbrc/9.3/28

[B37] Rakhonde G. Y. Panda S. Ahale S. R. Veratiya A. K. Deshkar A. M. Nayak L. (2024). “Role of microbes and organic amendments in stimulating ISR and SAR in plant disease management,” in Advances in Organic Farming (Palm Bay, FL: Apple Academic Press), 491–514.

[B38] Ramaiah N. Hill R. T. Chun J. Ravel J. Matte M. H. Straube W. L. . (2000). Use of a chiA probe for detection of chitinase genes in bacteria from the Chesapeake Bay. FEMS Microbiol. Ecol. 34, 63–71. doi: 10.1016/S0168-6496(00)00075-1 11053737

[B39] Rochlani A. Dalwani A. Shaikh N. Shaikh N. Sharma S. Saraf M. (2022). Plant growth promoting rhizobacteria as biofertilizers: application in agricultural sustainability. Acta Sci. Microbiol. 5, 12–21. doi: 10.31080/ASMI.2022.05.1028

[B40] Sahu P. K. Singh S. Gupta A. R. Gupta A. Singh U. B. Manzar N. . (2020). Endophytic *Bacilli* from medicinal-aromatic perennial Holy basil (*Ocimum tenuiflorum* L.) modulate plant growth promotion and induced systemic resistance against *Rhizoctonia solani* in rice (*Oryza sativa* L.). *Biol. Control* 150:104353. doi: 10.1016/j.biocontrol.2020.104353

[B41] Samjik M. K. S. (2024). Evaluation of bio-consortia against Fusarium wilt of tomato (Lycopersicon esculentum L.) (dissertation/master's thesis). Mahatma Phule Krishi Vidyapeeth, Rahuri, India.

[B42] Sandhya S. V. Preetha K. Vijayan K. K. (2017). Phylogenetic diversity of culturable bacteria in *Chaetoceros gracilis* mass culture system of a marine finfish hatchery. J. Mar. Biol. Assoc. India 59, 12–18. doi: 10.3354/ame01815

[B43] Senthilkumar M. Swarnalakshmi K. Govindasamy V. Lee Y. K. Annapurna K. (2009). Biocontrol potential of soybean bacterial endophytes against charcoal rot fungus, *Rhizoctonia bataticola*. Curr. Microbiol. 58, 288–293. doi: 10.1007/s00284-008-9329-z 19067044

[B44] Sessitsch A. Reiter B. Berg G. (2004). Endophytic bacterial communities of field-grown potato plants and their plant-growth-promoting and antagonistic abilities. Can. J. Microbiol. 50, 239–249. doi: 10.1139/w03-118 15213748

[B45] Shah D. Khan M. S. Aziz S. Ali H. Pecoraro L. (2021). Molecular and biochemical characterization, antimicrobial activity, stress tolerance, and plant growth-promoting effect of endophytic bacteria isolated from wheat varieties. Microorganisms 10:21. doi: 10.3390/microorganisms10010021 35056470 PMC8777632

[B46] Shanmugam V. Kanoujia N. (2011). Biological management of vascular wilt of tomato caused by *Fusarium oxysporum* f. sp. *lycospersici* by plant growth-promoting rhizobacterial mixture. Biol. Control 57, 85–93. doi: 10.1016/j.biocontrol.2011.02.001

[B47] Sharma S. Kumar P. Singh A. (2024). Systemic acquired resistance vs induced systemic resistance: a review. Agric. Rev. 45, 410–419. doi: 10.18805/ag.r-2411

[B48] Singh R. Pandey K. D. Singh M. Singh S. K. Hashem A. Al-Arjani A. B. F. . (2022). Isolation and characterization of endophytes bacterial strains of *Momordica charantia* L. and their possible approach in stress management. Microorganisms 10:290. doi: 10.3390/microorganisms10020290 35208743 PMC8877101

[B49] Singh S. Singh U. B. Malviya D. Paul S. Sahu P. K. Trivedi M. . (2020). Seed biopriming with microbial inoculant triggers local and systemic defense responses against *Rhizoctonia solani* causing banded leaf and sheath blight in maize (*Zea mays* L.). *Int. J. Environ. Res. Public Health* 17:1396. doi: 10.3390/ijerph17041396 PMC7068308 32098185

[B50] Singh U. B. Malviya D. Singh S. Pradhan J. K. Singh B. P. Roy M. . (2016). Bio-protective microbial agents from rhizosphere eco-systems trigger plant defense responses provide protection against sheath blight disease in rice (*Oryza sativa* L.). Microbiol. Res. 192, 300–312. doi: 10.1016/j.micres.2016.08.007 27664749

[B51] Souto A. L. Sylvestre M. Tölke E. D. Tavares J. F. Barbosa-Filho J. M. Cebrián-Torrejón G. (2021). Plant-derived pesticides as an alternative to pest management and sustainable agricultural production: prospects, applications and challenges. Molecules 26:4835. doi: 10.3390/molecules26164835 34443421 PMC8400533

[B52] Timofeeva A. M. Galyamova M. R. Sedykh S. E. (2023). Plant growth-promoting soil bacteria: nitrogen fixation, phosphate solubilization, siderophore production, and other biological activities. Plants 12:4074. doi: 10.3390/plants12244074 38140401 PMC10748132

[B53] Wang L. Fan R. Ma H. Sun Y. Huang Y. Wang Y. . (2023). Genomic and metabolomic insights into the antimicrobial compounds and plant growth-promoting potential of *Bacillus velezensis* Q-426. BMC Genomics 24:589. doi: 10.1186/s12864-023-09662-1 37794314 PMC10548584

[B54] Xia Y. Liu J. Chen C. Mo X. Tan Q. He Y. . (2022). The multi-functions and future prospects of endophytes and their metabolites in plant disease management. Microorganisms 10:1072. doi: 10.3390/microorganisms10051072 35630514 PMC9146654

[B55] Yang R. Fan X. Cai X. Hu F. (2015). The inhibitory mechanisms by mixtures of two endophytic bacterial strains isolated from *Ginkgo biloba* against pepper phytophthora blight. Biol. Control 85, 59–67. doi: 10.1016/j.biocontrol.2014.09.013

[B56] Yuan J. Raza W. Shen Q. Huang Q. (2012). Antifungal activity of Bacillus amyloliquefaciens NJN-6 volatile compounds against Fusarium oxysporum f. sp. cubense. Appl Environ. Microbiol. 78, 5942–5944. doi: 10.1128/aem.01357-12 22685147 PMC3406121

[B57] Zhang X. Tong J. Dong M. Akhtar K. He B. (2022). Isolation, identification and characterization of nitrogen fixing endophytic bacteria and their effects on cassava production. PeerJ 10:e12677. doi: 10.7717/peerj.12677 35127278 PMC8796710

