# Supplementary material for: Biocontrol potential of endophytic bacteria against Fusarium wilt pathogen (Fusarium oxysporum f. sp. ciceris) in chickpea
Source: Front Microbiol. 2026 May 28;17:1787705. doi: 10.3389/fmicb.2026.1787705 (PMC13254275; doi:10.3389/fmicb.2026.1787705)
Supplement: Supplementary file 1 [file Supplementary_file_1.docx]

**Supplementary Material Tables**

**Table 1. List of total isolated endophytic bacteria**

| **Sl. No.** | **Source** | **Plant Part** | **Isolates** | **No. of isolates** |
| --- | --- | --- | --- | --- |
| 1 | Rice | Root | RLB E-1, RLB E-2, RLB E-3, RLBE-4 | 4 |
| 2 | Rice | Leaf | RLB E- 5, RLB E-6 | 2 |
| 3 | Rice | Stem | RLB E-7, RLB E-8 | 2 |
| 4 | Chickpea | Root | RLB E-9, RLB E-10, RLB E-56, RLB E-57 | 4 |
| 5 | Chickpea | Stem | RLB E-11, RLB E-12, RLB E-13, RLB E-14 | 4 |
| 6 | Chickpea | Leaf | RLB E-15, RLB E-16, RLB E-17, RLB E-18, RLB E-19 | 5 |
| 7 | Taro | Root | RLB E-20 | 1 |
| 8 | Calotropis | Root | RLB E-21 | 1 |
| 9 | Lawn grass | Root | RLB E-22, RLB E-23, RLB E-43, RLB E-44 | 4 |
| 10 | Mungbean | Leaf | RLB E-24, RLB E-25, RLB E-26, | 3 |
| 11 | Mungbean | Root | RLB E-27 | 1 |
| 12 | Spinach | Leaf | RLB E-28, RLB E-29, RLB E-30, RLB E-31 | 4 |
| 13 | Bean | Leaf | RLB E-32, RLB E-33, RLB E-34 | 3 |
| 14 | Bean | Root | RLB E-35, RLB E-36, RLB E-37, RLB E-38 | 4 |
| 15 | Bean | Stem | RLB E-39, RLB E-40, RLB E-41, RLB E-42 | 4 |
| 16 | Mustard | Root | RLB E-45, RLB E-46, RLB E-47, RLB E-48, RLB E-49 | 5 |
| 17 | Mustard | Leaf | RLB E-50, RLB E-51, RLB E-52, RLB E-53 | 4 |
| 18 | Mustard | Stem | RLB E-54, RLB E-55 | 2 |
| 19 | Okra | Root | RLB E-58, RLB E-59 | 2 |
| 20 | Tomato l | Leaf | RLB E-60, RLB E-61 | 2 |
| 21 | Onion | Leaf | RLB E-62, RLB E-63, RLB E-64, RLB E-65 | 4 |
| 22 | Onion | Root | RLB E-66, RLB E-67 | 2 |
| 23 | Wheat | Stem | RLB E-68, RLB E-69, RLB E-70 | 3 |
| 24 | Wheat | Root | RLB E-71, RLB E-72, RLB E-73 | 3 |
| **Total isolates** | | | | **73** |

**Table 2: Morphological Characterization of effective EB isolates**

| **Isolate Code** | **Shape** | **Size** | **Colour** | **Margin** | **Gram stain** |
| --- | --- | --- | --- | --- | --- |
| RLB E - 1 | Circular | Small | Creamish | Smooth | Negative |
| RLB E - 25 | Irregular | Large | Creamish | Rough | Positive |
| RLB E - 30 | Circular | Small | Creamish | Smooth | Negative |
| RLB E - 33 | Circular | Small | Creamish | Smooth | Positive |
| RLB E - 64 | Circular | Small | Creamish | Smooth | Negative |

**Table 3: Biochemical and Functional Characterization of EB Isolates**

| **Isolate Code** | **Oxidase** | **Catalase** | **KOH** | **Gram staining** | **Amylase** | **Cellulase** | **Chitinase** | **Motility** | **Phosphate solubulization** | **Protease** | **IAA Production** |
| --- | --- | --- | --- | --- | --- | --- | --- | --- | --- | --- | --- |
| RLB E-1 | + | + | - | + | - | - | - | + | - | - | ++ |
| RLB E-25 | + | + | - | + | + | - | - | + | + | + | - |
| RLB E-30 | - | - | + | - | + | - | - | + | - | + | - |
| RLB E-33 | + | + | + | - | + | - | - | + | + | + | - |
| RLB E-64 | + | + | - | + | + | - | - | + | + | + | - |

**Table 4: Biochemical and Functional Characterization of EB Isolates by kit method**

| **Test No.\Isolate Code** | **RLB E-1** | **RLB E-25** | **RLB E-30** | **RLB E-33** | **RLB E-64** |
| --- | --- | --- | --- | --- | --- |
| ONPG | - | - | - | - | - |
| Lysine utilization | + | + | + | - | - |
| Ornithine utilization | + | + | - | - | - |
| Urease | ++ | - | - | + | - |
| Phenylalanine deamination | - | - | - | + | - |
| Nitrate reduction | - | + | - | + | + |
| H_2_S production | + | - | - | ++ | - |
| Citrate utilization | + | - | + | + | - |
| Voges Proskauer’s | - | ++ | ++ | + | ++ |
| Methyl Red | - | + | + | - | + |
| Indole | - | - | - | - | - |
| Malonate utilization | + | - | - | + | - |
| Esculin hydrolysis | + | + | ++ | ++ | ++ |
| Arabinose | - | - | - | - | - |
| Xylose | - | - | + | - | - |
| Adonitol | - | - | - | - | + |
| Rhamnose | - | - | - | - | + |
| Cellobiose | + | + | ++ | - | ++ |
| Melibiose | + | - | - | - | - |
| Saccharose | + | + | ++ | + | ++ |
| Rafinose | + | - | + | - | - |
| Trehalose | - | + | + | + | + |
| Glucose | - | ++ | ++ | - | ++ |
| Lactose | - | - | - | - | - |
| Oxidase | + | + | - | + | + |

**Table 5: Thermostability tolerance of EB against FOC**

| **Isolate** | **40**°C | **50**°C | **60**°C | **70**°C | **80**°C | **90**°C |
| --- | --- | --- | --- | --- | --- | --- |
| RLB E-1 | 30.0 | 48.5 | 52.5 | 40.5 | 38.0 | 58.5 |
| RLB E-25 | 25.0 | 38.0 | 23.5 | 25.0 | 53.5 | 53.5 |
| RLB E-30 | 37.5 | 56.0 | 55.5 | 56.0 | 42.0 | 45.5 |
| RLB E-33 | 33.5 | 44.5 | 40.0 | 44.5 | 50.0 | 56.0 |
| RLB E-64 | 27.5 | 48.0 | 47.0 | 41.5 | 48.0 | 47.0 |
| C.D. | 1.56 | 1.46 | 1.39 | 1.32 | 1.52 | 1.43 |
| SE (m) | 0.49 | 0.46 | 0.43 | 0.41 | 0.47 | 0.44 |

**Table 6: Antibiotic and Lipopeptide genes analysis of effective endophytic bacteria**

| **Isolate** | **Chit-A** | **Chitinase** | **Surfactin** | **Iturin-D** | **Fengymycin-D** | **Bam-C** |
| --- | --- | --- | --- | --- | --- | --- |
| RLB E- 1 | - | - | - | - | - | + |
| RLB E- 25 | + | - | - | + | - | + |
| RLB E- 30 | + | - | - | - | - | + |
| RLB E- 33 | + | - | + | - | - | - |
| RLB E- 64 | + | - | - | - | - | + |

**Table 7. Pot-Based Efficacy Assessment of an Antagonistic Bacterial Strain Against *Fusarium* Wilt of Chickpea (SEASON-1)**

| **Treatment No.** | **Treatment Details** | **Germination %** | **Disease Intensity** | | | **Mortality** | | | |
| --- | --- | --- | --- | --- | --- | --- | --- | --- | --- |
|  |  |  | **15 DAS** | **30 DAS** | **45 DAS** | **Pre-emergence mortality** | **Post-emergence mortality** | | **Total Mortality** |
| T1 | RLB E – 25 Seed Priming | 35.56 | 24.44 | 31.11 | 37.78 | 64.21 | | 10.15 | 74.36 |
| T2 | RLB E – 33 Seed Priming | 37.78 | 13.33 | 17.78 | 33.33 | 62.22 | | 8.61 | 70.83 |
| T3 | RLB E – 30 Seed Priming | 33.33 | 20.00 | 28.89 | 40.00 | 66.67 | | 12.50 | 79.17 |
| T4 | RLB E – 64 Seed Priming | 37.78 | 17.78 | 24.45 | 37.78 | 62.22 | | 12.27 | 74.49 |
| T5 | RLB E – 1 Seed Priming | 28.89 | 24.45 | 33.33 | 42.22 | 71.11 | | 13.55 | 84.66 |
| T6 | Chemical Control Seed Priming | 71.11 | 11.11 | 15.55 | 17.78 | 28.89 | | 12.70 | 41.59 |
| T7 | Control (Infected Soil) | 22.22 | 28.89 | 48.89 | 68.89 | 77.78 | | 6.14 | 83.92 |
| T8 | Control (Healthy Soil) | 73.33 | 0.00 | 6.67 | 15.55 | 26.67 | | 14.87 | 41.54 |
| T9 | RLB E – 25 Drenching | 46.67 | 15.55 | 22.22 | 28.89 | 53.33 | | 19.11 | 72.44 |
| T10 | RLB E – 33 Drenching | 62.22 | 4.45 | 11.11 | 20.00 | 37.55 | | 16.07 | 53.62 |
| T11 | RLB E – 30 Drenching | 44.45 | 15.55 | 20.00 | 31.11 | 55.32 | | 15.52 | 70.84 |
| T12 | RLB E – 64 Drenching | 57.78 | 11.11 | 15.55 | 22.22 | 41.99 | | 21.23 | 63.22 |
| T13 | RLB E – 1 Drenching | 37.78 | 17.78 | 22.22 | 33.33 | 61.99 | | 15.06 | 77.05 |
| T14 | Chemical Control Drenching | 66.66 | 6.67 | 20.00 | 20.00 | 33.11 | | 14.13 | 47.24 |
| T15 | RLB E – 25 Seed Priming + Drenching | 77.78 | 11.11 | 13.33 | 15.55 | 21.99 | | 14.36 | 36.35 |
| T16 | RLB E – 33 Seed Priming + Drenching | 95.55 | 0.00 | 6.67 | 8.89 | 4.45 | | 5.73 | 10.18 |
| T17 | RLB E – 30 Seed Priming + Drenching | 57.78 | 15.55 | 17.78 | 24.45 | 41.99 | | 16.96 | 58.95 |
| T18 | RLB E – 64 Seed Priming + Drenching | 91.11 | 8.89 | 15.55 | 15.55 | 8.66 | | 10.57 | 19.23 |
| T19 | RLB E – 1 Seed Priming + Drenching | 57.78 | 15.55 | 22.22 | 26.67 | 41.99 | | 18.25 | 60.24 |
| T20 | Chemical Control Seed Priming + Drenching | 93.33 | 4.45 | 8.89 | 11.11 | 6.44 | | 12.50 | 18.94 |
| C.D. | | 27.159 | 8.00 | 8.20 | 8.80 | 2.81 | | 3.68 |  |
| SE (m) | | 9.467 | 2.78 | 2.85 | 3.06 | 0.98 | | 1.28 |  |

**Table 8. Pot-Based Efficacy Assessment of an Antagonistic Bacterial Strain Against *Fusarium* Wilt of Chickpea (SEASON-2)**

| **Treatment No.** | **Treatment Details** | **Germination %** | **Disease Intensity** | | | **Mortality** | | | |
| --- | --- | --- | --- | --- | --- | --- | --- | --- | --- |
|  |  |  | **15**  **DAS** | **30**  **DAS** | **45**  **DAS** | **Pre-emergence mortality** | **Post-emergence mortality** | | **Total Mortality** |
| T1 | RLB E – 25 Seed Priming | 58.89 | 17.78 | 31.11 | 42.22 | 41.11 | | 10.24 | 51.35 |
| T2 | RLB E – 33 Seed Priming | 51.11 | 15.56 | 22.22 | 35.553 | 48.89 | | 12.93 | 61.82 |
| T3 | RLB E – 30 Seed Priming | 53.33 | 28.89 | 28.89 | 35.553 | 46.67 | | 10.39 | 57.06 |
| T4 | RLB E – 64 Seed Priming | 72.22 | 20.00 | 31.11 | 35.553 | 27.78 | | 7.81 | 35.59 |
| T5 | RLB E – 1 Seed Priming | 51.11 | 28.89 | 35.55 | 44.447 | 48.89 | | 10.07 | 58.96 |
| T6 | Chemical Control Seed Priming | 79.00 | 8.89 | 13.33 | 15.553 | 21 | | 14.23 | 35.23 |
| T7 | Control (Infected Soil) | 58.89 | 31.11 | 51.11 | 73.333 | 41.11 | | 9.93 | 51.04 |
| T8 | Control (Healthy Soil) | 84.44 | 6.67 | 13.33 | 17.777 | 15.56 | | 6.47 | 22.03 |
| T9 | RLB E – 25 Drenching | 49.89 | 13.33 | 20.00 | 31.113 | 50.11 | | 12.32 | 62.43 |
| T10 | RLB E – 33 Drenching | 77.78 | 11.11 | 17.78 | 24.447 | 22.22 | | 14.57 | 36.79 |
| T11 | RLB E – 30 Drenching | 66.67 | 17.78 | 20.00 | 31.11 | 33.33 | | 15.56 | 48.89 |
| T12 | RLB E – 64 Drenching | 75.56 | 8.89 | 20.00 | 24.443 | 24.44 | | 11.44 | 35.88 |
| T13 | RLB E – 1 Drenching | 64.45 | 15.55 | 24.45 | 31.11 | 35.55 | | 18.21 | 53.76 |
| T14 | Chemical Control Drenching | 82.22 | 11.11 | 13.33 | 20 | 17.78 | | 13.74 | 31.52 |
| T15 | RLB E – 25 Seed Priming + Drenching | 84.44 | 4.45 | 13.33 | 17.777 | 15.56 | | 7.09 | 22.65 |
| T16 | RLB E – 33 Seed Priming + Drenching | 91.11 | 0.00 | 4.45 | 6.67 | 8.89 | | 4.59 | 13.48 |
| T17 | RLB E – 30 Seed Priming + Drenching | 71.11 | 13.33 | 15.55 | 26.67 | 28.89 | | 14.44 | 43.33 |
| T18 | RLB E – 64 Seed Priming + Drenching | 88.89 | 2.22 | 6.67 | 13.33 | 11.11 | | 5.35 | 16.46 |
| T19 | RLB E – 1 Seed Priming + Drenching | 71.11 | 13.33 | 17.78 | 31.11 | 28.89 | | 11.54 | 40.43 |
| T20 | Chemical Control Seed Priming + Drenching | 91.11 | 0.00 | 6.67 | 13.33 | 8.89 | | 5.59 | 14.48 |
| C.D. | | 23.61 | 9.56 | 7.40 | 8.80 | 4.96 | | 5.33 |  |
| SE (m) | | 8.23 | 3.32 | 2.57 | 3.06 | 1.73 | | 1.85 |  |

**Table 9: Field-Based Efficacy Assessment of Antagonistic Bacterial Strains Against Chickpea *Fusarium* Wilt**

| **Treatment No.** | **Treatment Details** | **Germination %** | **Disease Intensity** | | | **Yield (t/ha)** |
| --- | --- | --- | --- | --- | --- | --- |
|  |  |  | **15DAS** | **30DAS** | **45DAS** |  |
| T1 | RLB E – 25 Seed Priming | 82.97 | 49.77 | 46.00 | 18.56 | 2.21 |
| T2 | RLB E – 25 Drenching | 86.19 | 58.78 | 53.44 | 21.67 | 2.18 |
| T3 | RLB E – 25 Seed Priming + Drenching | 93.57 | 48.00 | 42.00 | 18.44 | 2.29 |
| T4 | RLB E – 33 Seed Priming | 86.19 | 39.89 | 36.44 | 16.22 | 2.36 |
| T5 | RLB E – 33 Drenching | 89.52 | 32.44 | 31.11 | 20.11 | 2.47 |
| T6 | RLB E – 33 Seed Priming + Drenching | 91.67 | 36.67 | 29.44 | 15.11 | 2.48 |
| T7 | RLB E – 30 Seed Priming | 81.90 | 61.22 | 58.00 | 25.00 | 2.05 |
| T8 | RLB E – 30 Drenching | 91.43 | 58.44 | 54.89 | 26.00 | 2.00 |
| T9 | RLB E – 30 Seed Priming + Drenching | 89.76 | 63.11 | 58.67 | 24.00 | 2.16 |
| T10 | RLB E – 64 Seed Priming | 88.93 | 39.33 | 35.56 | 19.22 | 2.31 |
| T11 | RLB E – 64 Drenching | 89.05 | 36.00 | 32.56 | 24.44 | 2.29 |
| T12 | RLB E – 64 Seed Priming + Drenching | 90.36 | 39.11 | 38.89 | 18.56 | 2.33 |
| T13 | Chemical Control Seed Priming | 94.17 | 36.44 | 26.33 | 17.33 | 2.55 |
| T14 | Chemical Control Drenching | 92.86 | 26.33 | 24.67 | 18.00 | 3.01 |
| T15 | Chemical Control Seed Priming + Drenching | 94.76 | 33.67 | 21.33 | 15.56 | 3.22 |
| T16 | Control | 51.53 | 68.44 | 64.67 | 46.44 | 1.47 |
| T17 | RLB E – 1 Drenching | 59.40 | 66.11 | 61.00 | 32.78 | 1.59 |
| T18 | RLB E – 1 Seed Priming + Drenching | 68.69 | 64.56 | 61.78 | 34.22 | 1.51 |
| T19 | RLB E – 1 Seed Priming | 70.36 | 65.56 | 64.33 | 30.00 | 1.80 |
| C.D. | | 7.03 | 8.05 | 19.95 | 2.60 | 0.77 |
| SE (m) | | 2.44 | 2.79 | 6.929 | 0.90 | 0.26 |

**Supplementary Material Photos**

**Amylase test Protease test Phosphate solubilization**


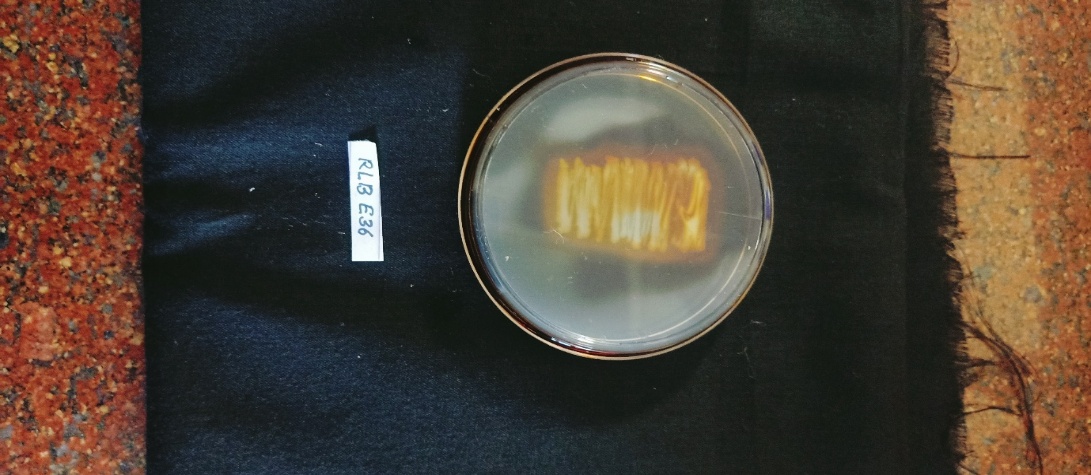

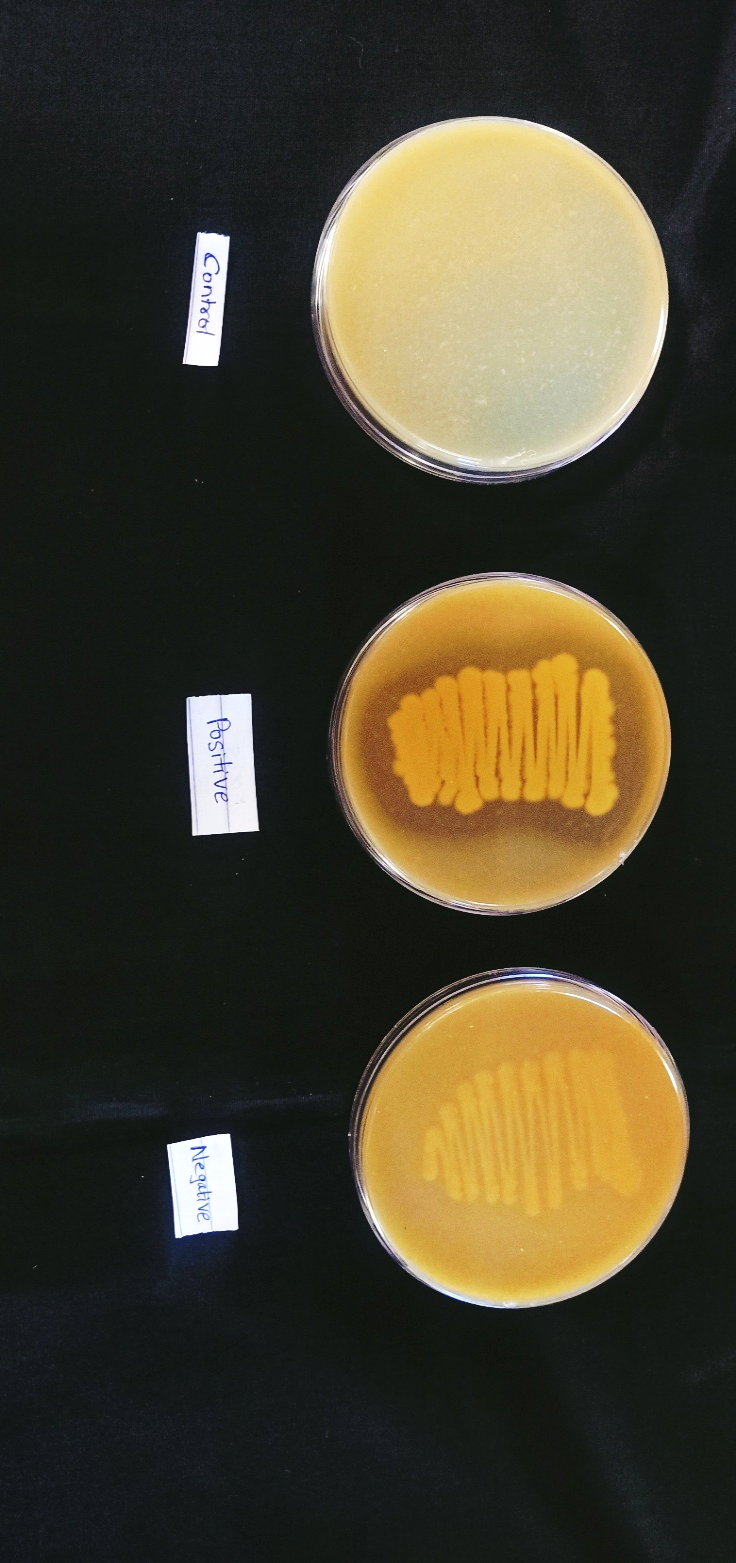

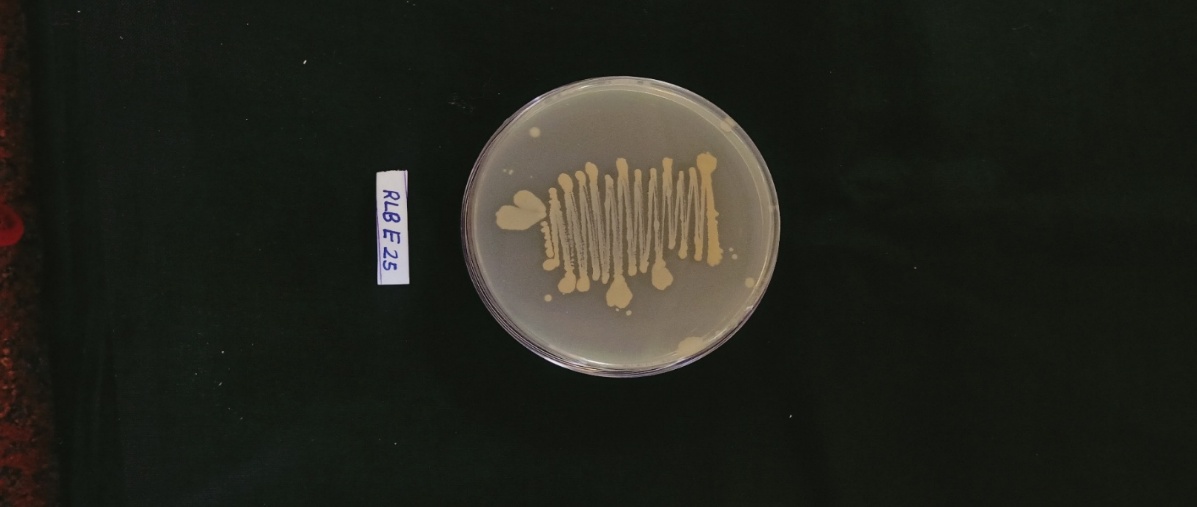


**Cellulase test Chitinase test Oxidase test**


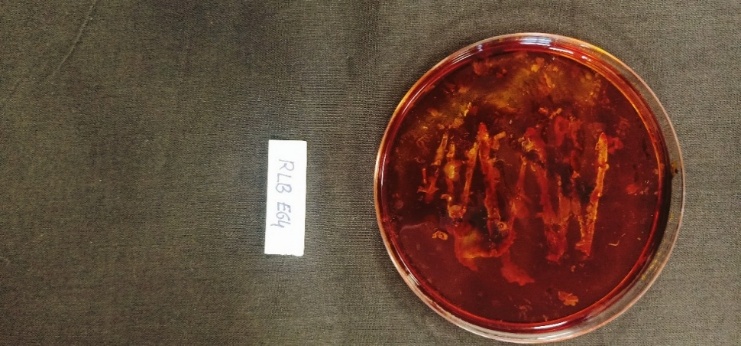

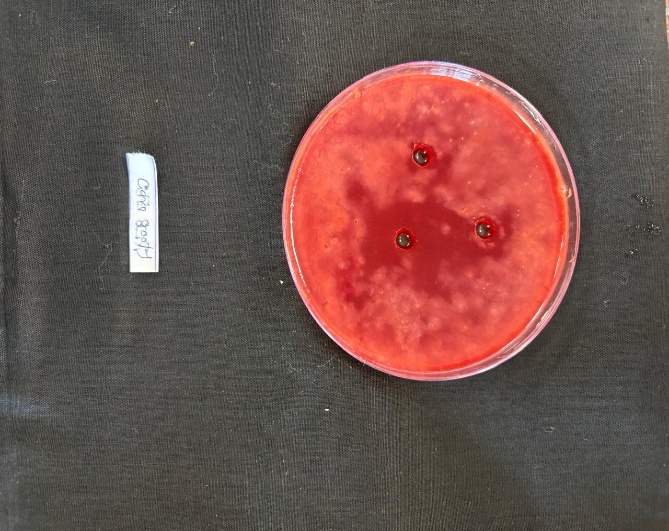

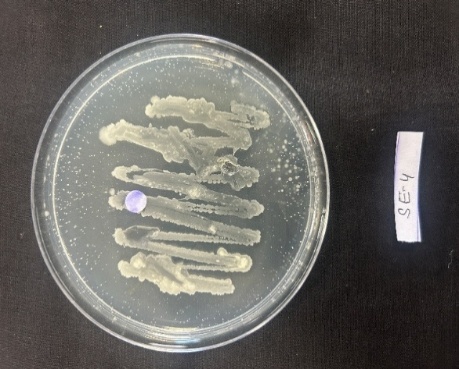


**Catalase test KOH TEST IAA production test**


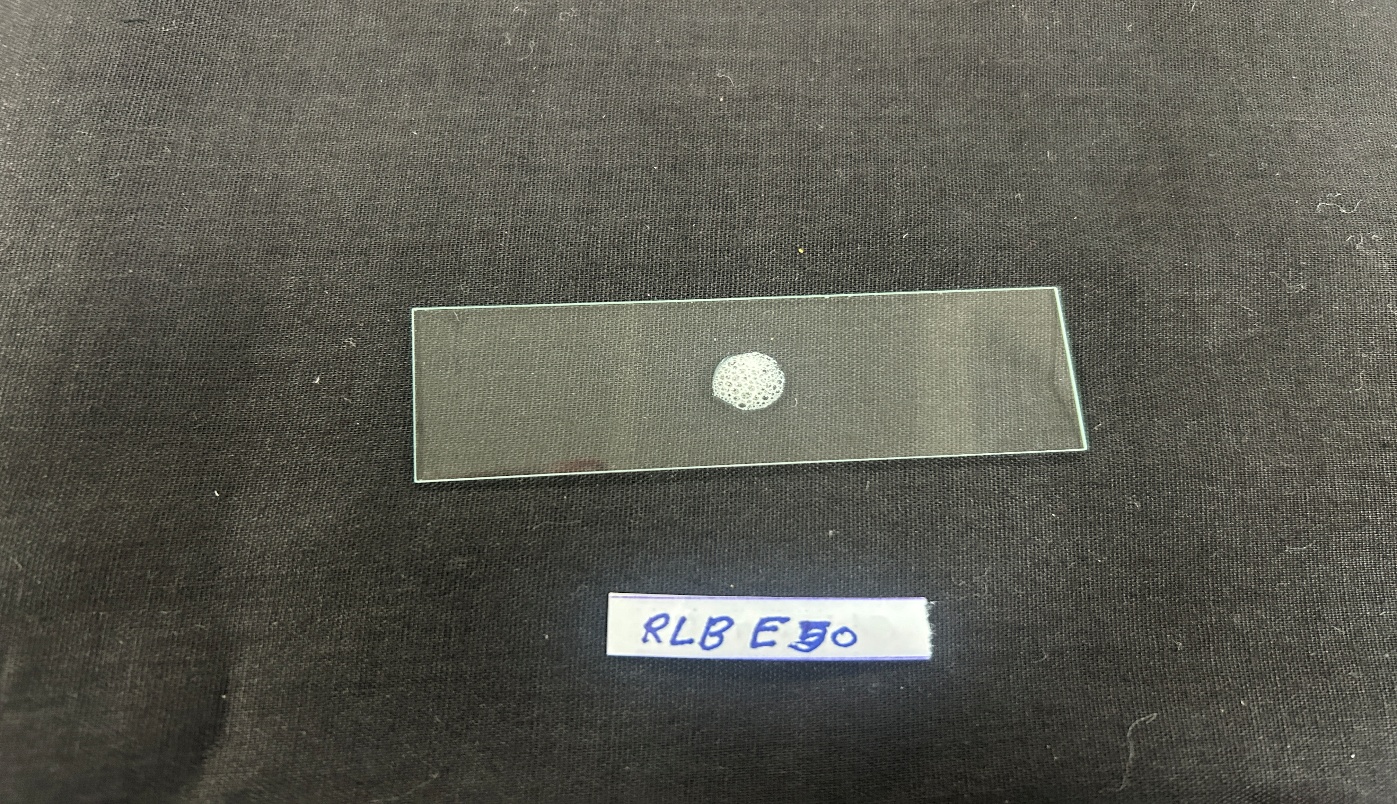

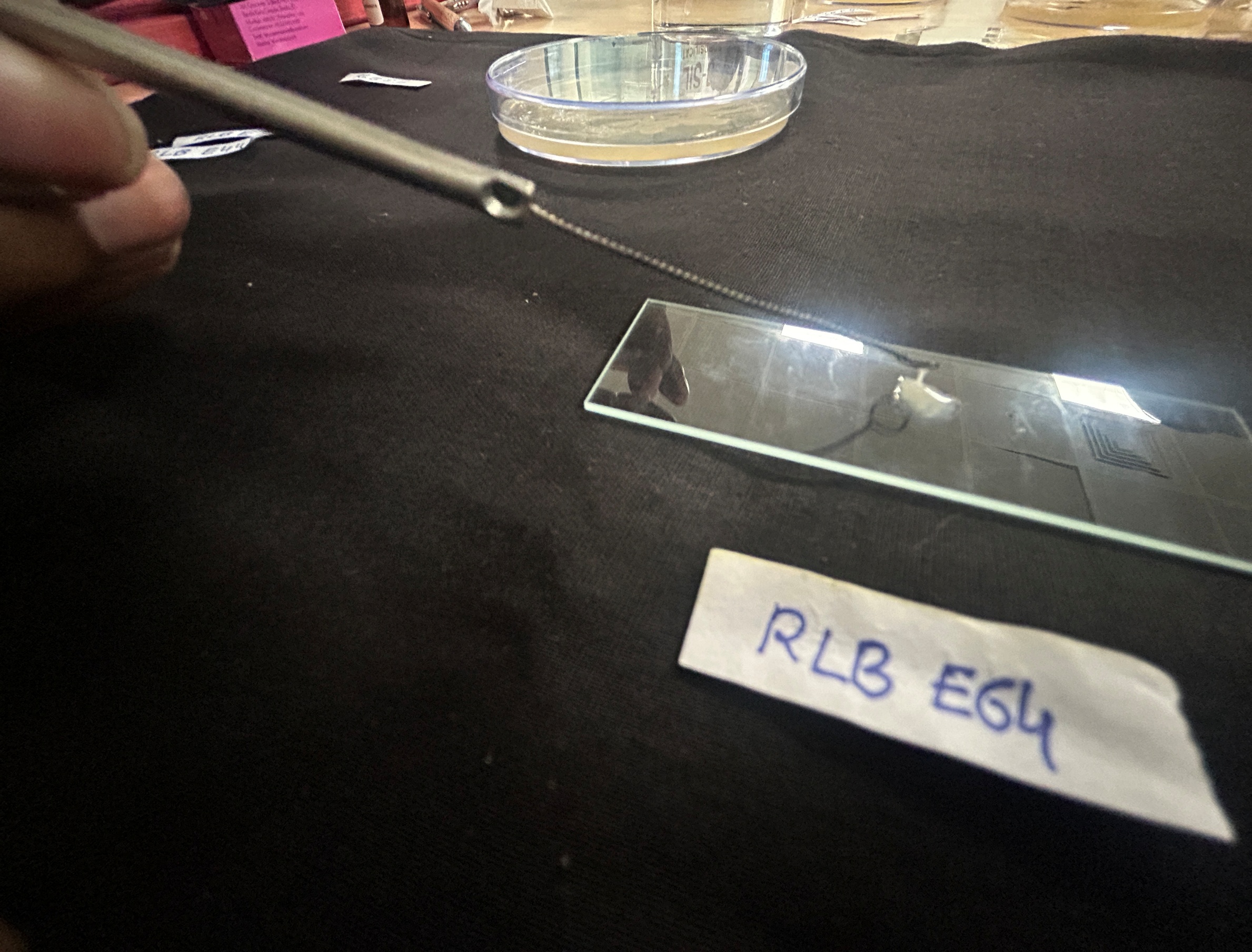

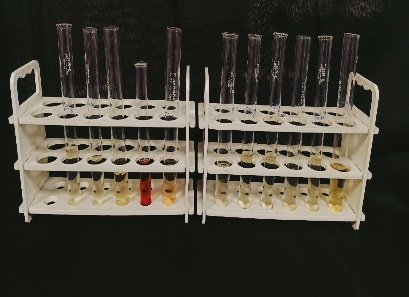


**Fig 1. Biochemical Characterization of bacterial endophytes**


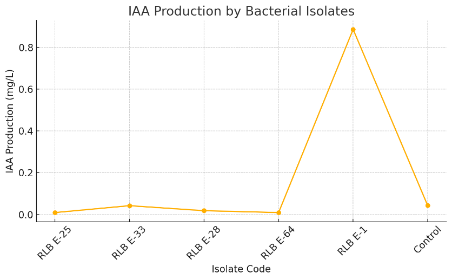

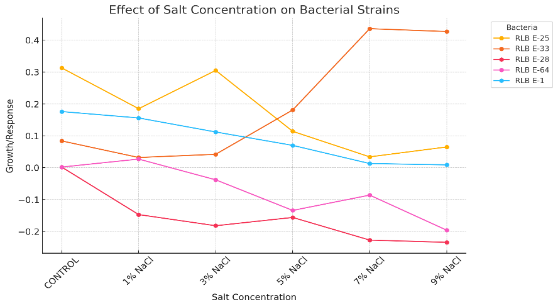

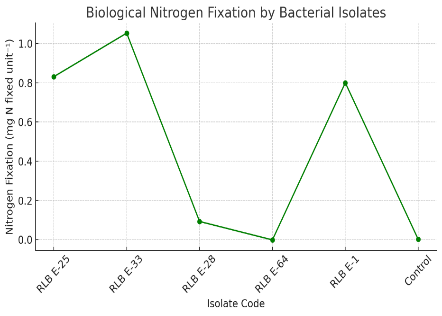


1. **IAA Production b. Salt tolerance c. BNF**

**Fig 2. Graphical Representation of Endophytic Bacteria for IAA Production, Salt Tolerance, and Biological Nitrogen Fixation (BNF)**

**Pot Study-Season 1 Pot Study-Season 2 Field management**


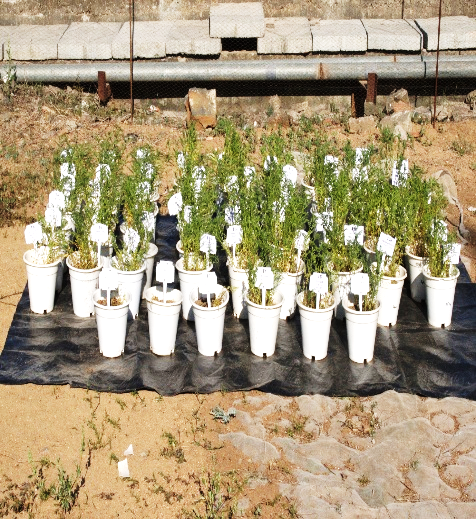

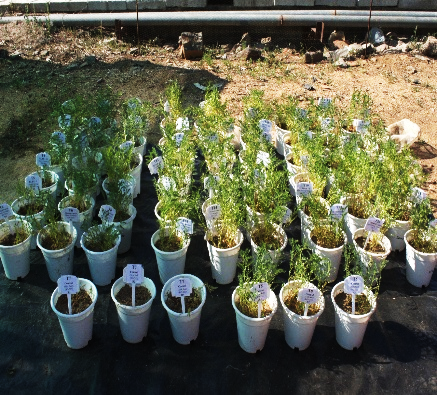

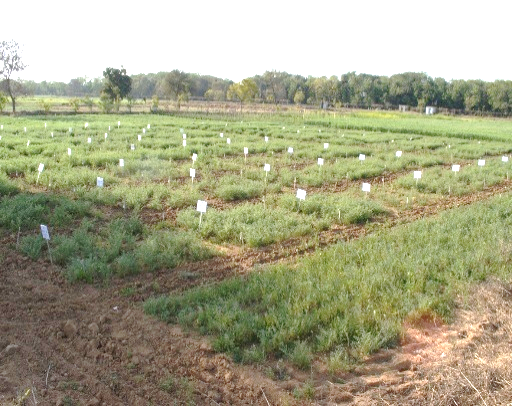


**Fig 3. Pot and Field efficacy assessment of the antagonistic bacterium against *Fusarium* wilt of chickpea**
